# Identification and Expression of Fructose-1,6-Bisphosphate Aldolase Genes and Their Relations to Oil Content in Developing Seeds of Tea Oil Tree (*Camellia oleifera*)

**DOI:** 10.1371/journal.pone.0107422

**Published:** 2014-09-12

**Authors:** Yanling Zeng, Xiaofeng Tan, Lin Zhang, Nan Jiang, Heping Cao

**Affiliations:** 1 Key Laboratory of Cultivation and Protection for Non-Wood Forest Trees of Ministry of Education, Central South University of Forestry and Technology, Changsha, Hunan, China; 2 Key Laboratory of Non-Wood Forest Products of State Forestry Administration, Central South University of Forestry and Technology, Changsha, Hunan, China; 3 Key Laboratory of Green Packaging and Biological Nanotechnology, Hunan University of Technology, Zhuzhou, Hunan, China; 4 U.S. Department of Agriculture, Agricultural Research Service, Southern Regional Research Center, New Orleans, Louisiana, United States of America; University Paris South, France

## Abstract

Tea oil tree (*Camellia oleifera*, Co) provides a fine edible oil source in China. Tea oil from the seeds is very beneficial to human health. Fructose-1,6-bisphosphate aldolase (FBA) hydrolyzes fructose-1,6-bisphosphate into dihydroxyacetone phosphate and glyceraldehyde 3-phosphate, two critical metabolites for oil biosynthesis. The objectives of this study were to identify FBA genes and investigate the relationship between FBA gene expression and oil content in developing seeds of tea oil tree. In this paper, four developmentally up-regulated *CoFBA* genes were identified in *Camellia oleifera* seeds based on the transcriptome from two seed developmental stages corresponding to the initiation and peak stages of lipid biosynthesis. The expression of *CoFBA* genes, along with three key oil biosynthesis genes *CoACP*, *CoFAD2* and *CoSAD* were analyzed in seeds from eight developmental stages by real-time quantitative PCR. The oil content and fatty acid composition were also analyzed. The results showed that *CoFBA* and *CoSAD* mRNA levels were well-correlated with oil content whereas *CoFAD2* gene expression levels were correlated with fatty acid composition in *Camellia* seeds. We propose that *CoFBA* and *CoSAD* are two important factors for determining tea oil yield because *CoFBA* gene controls the flux of key intermediates for oil biosynthesis and *CoSAD* gene controls the synthesis of oleic acid, which accounts for 80% of fatty acids in tea oil. These findings suggest that tea oil yield could be improved by enhanced expression of *CoFBA* and *CoSAD* genes in transgenic plants.

## Introduction

*Camellia oleifera* (Co, tea oil tree) provides high quality edible oil. This small tree is originated and widely cultivated in China. Tea oil (*Camellia* oil) from the seeds is a sweetish seasoning and cooking oil with over 80% monounsaturated fatty acids. The seed residue after oil extraction has been widely used for laundry purposes in rural areas. Tea oil is sold as cooking oil in supermarkets throughout China as well as Australia, New Zealand and the United States.

Tea oil is very beneficial to human health. Tea oil can lower cholesterol, decrease lipid concentration and prevent hypertension and hardening of arteries [1]–[3]. Tea oil has anti-microbial and antioxidant properties and known to induce cell cycle arrest and apoptosis in cancerous cell lines [1], [4], [5]. Tea oil has also been shown to have antiulcer effects against ketoprofen-induced oxidative damage in the stomach and intestine [6]. Flavonoids from seed shells have analgesic and anti-inflammatory effects [7], [8]. Defattened seeds of the plant have saponins with anti-inflammatory properties [2]. However, the development of tea oil industry is limited due to the low oil yield of tea oil tree. Most of the early research efforts were focused on fatty acid analysis of the seeds. In order to improve tea oil production, many genes coding for key enzymes in tea oil biosynthesis pathway have been identified in the tree [9]–[14].

Fructose-1,6-bisphosphate aldolase (FBA, EC4.1.2.13, or simply aldolase) is a key enzyme in the glycolytic pathway. FBA catalyzes a reversible reaction by converting fructose-1,6-bisphosphate (FBP) into dihydroxyacetone phosphate (DHAP) and glyceraldehyde 3-phosphate (G3P) [15]. DHAP and G3P are two key intermediates for oil biosynthesis. DHAP is converted to glycerol-3-phosphate by glycerol phosphate dehydrogenase [16], which is used to generate phosphatidic acid by the action of acyltransferases. Phosphatidic acid is then hydrolyzed by phosphatidic acid phosphatase to produce diacylglycerol (DAG), the key substrate of diacylglycerol acyltransferease (DGAT) for the synthesis of triacylglycerols (TAGs) [17], [18]. Meanwhile, G3P is converted to pyruvate by multiple enzymatic reactions, which is used to generate acetyl-CoA. Acetyl-CoA is converted to malonyl CoA by acetyl-CoA carboxylase. Malonyl CoA is then utilized for the synthesis of fatty acids, the other key element for oil biosynthesis [19]. Therefore, FBA not only affects fatty acid synthesis but also provides acylglycerol for oil biosynthesis.

The objectives of this study were to identify *FBA* genes, analyze the expression patterns of these genes and investigate the relationship between *FBA* gene expression and oil content/fatty acid composition in developing seeds of tea oil tree. In this paper, the full-length cDNAs for *CoFBA1, CoFBA2, CoFBA3* and *CoFBA4* genes were identified from *Camellia oleifera* based on the analysis of its seed transcriptome digital library. Using real-time quantitative PCR (RT-qPCR), the expression of *CoFBA* genes was quantified using RNA from eight development stages of seeds. Expression profiles of three key fatty acid synthesis genes coding for acyl carrier protein (*CoACP*) [11], stearoyl-ACP desaturase (*CoSAD*) [13] and oleate desaturase (*CoFAD2*) [20] were also analyzed in the seeds. We further analyzed the oil content and fatty acid composition of the seeds from eight developmental stages. Our results showed that *CoFBA* as well as *CoSAD* gene expression was well-correlated with oil content in *Camellia* seeds.

## Materials and Methods

### Ethics Statement

No specific permits were required from collecting the samples because the trees were public-owned and the field studies did not involve protected species.

### Plant Materials and RNA Isolation

*Camellia oleifera* var. ‘Hua shuo’ was used for this study. Eight stages of seeds were collected for gene expression analysis (May 5, June 5, July 4, August 4, September 4, September 11, September 26 and October 24, 2010). Eight different stages of seeds were used for oil content and fatty acid composition analysis (August 25, September 4, September 11, September 19, September 26, October 3, October 10 and October 24, 2010). RNA from the seeds collected on June 5 and October 24 was used to construct transcriptome libraries. Total RNA was isolated from the seeds with PureLink RNA Mini Kit according to the manufacturer's instruction (Invitrogen, USA). The potential genomic DNA contamination in the RNA samples was eliminated by RNase-free DNaseI digestion (Fermentas, Canada).

### cDNA library Construction

Poly-A mRNA was purified from total RNA isolated from the June 5 and October 24 seeds using oligo (dT) magnetic beads and fragmented into 200–500 bp pieces using divalent cations. The mRNA fragments were reverse transcribed into first-strand cDNA using SuperScript II reverse transcriptase and random primers (Life Technologies). The second-strand cDNA was synthesized by *E. coli* DNA polymerase I (Invitrogen, USA). After double-stranded cDNA synthesis, fragments were end repaired and A-tailed. The final cDNA library was created by purifying and enriching the double-stranded cDNA with PCR.

### Transcriptome Sequencing and Unigene Analysis

The cDNA sequences were determined through a paired-end flow cell using HiSeq 2000 platform (Beijing Genomics Institute, Shenzhen, China). The clean reads after sequencing were de novo assembled using Trinity with default K-mers  = 25 [21]. The contigs without ambiguous bases were obtained by conjoining the K-mers in an unambiguous path. The clean reads were mapped back to contigs using Trinity to construct unigenes with the paired-end information. Finally, the contigs were connected with Trinity, and sequences that could not be extended on either end are defined as unigenes for each library. Unigene sequences were aligned with those in the online database NR, SwissProt, Kyoto Encyclopedia of Genes and Genomes (KEGG) and Cluster of Orthologous Group of proteins (COG). Unigenes were categorized into gene ontologies (GO) and functionally annotated using Blast2GO software [22]. Unigenes with p-value <0.00001 were regarded as known genes [23]. Pathway enrichment analysis of unigenes was performed using KEGG database. FBA genes were identified from the unigenes for digital expression profile analysis [24].

### Identification and Digital Analysis of FBA Gene Expression

*CoFBA* genes were identified from the unigenes for digital expression profile analysis [24]. NCBI's Blast program was used to identify *CoFBA* genes from the *Camellia oleifera* seed transcriptome libraries based on sequence conservation to *FBA* genes from other plants. The full-length cDNAs of *CoFBA* genes were obtained from cDNA libraries by PCR using RACE technique. To compare differences in *FBA* gene expression, tag frequencies of both libraries were analyzed by IDEG6 software (http://telethon.bio.unipd.it/bioinfo/IDEG6/) according to the method described by Audic and Claverie [24]. The false discovery rate (FDR) was used to determine the threshold P-value for multiple testing. Calculation of unigene expression uses the reads per kb per million reads (RPKM) method [25]. FDR<0.001 and absolute value of the log2 RPKM ratio >1 were used as the threshold to determine significant differences in gene expression.

### Quantitative Gene Expression Analysis

Genes coding for several CoFBA and three oil biosynthetic enzymes/proteins (CoACP, CoFAD2 and CoSAD) were selected for quantitative expression analysis by RT-qPCR. ACP, SAD and FAD were selected for comparative studies and used as positive controls related to oil biosynthesis in the seeds because they are known components in oil biosynthesis pathway. Based on earlier experimental results [26], *CoGAPDH* gene was used as the reference gene for quantitative gene expression by RT-qPCR. The names, GenBank accession numbers and primer sequences of the genes for RT-qPCR assays are showed in Table 1. The relative abundance of mRNAs at different developmental stages was analyzed by CFX manager software (Bio-Rad, USA). RT-qPCR was performed in triplicates by SYBR Green qPCR assay essentially as described [27] with 10 µM each of the forward and reverse primers using the following PCR program: initial denaturation at 95°C for 5 min, followed by 40 cycles of 95°C for 10 sec and 55°C for 30 s. The relative gene expression for RT-qPCR data was calculated by the 2(-Delta Delta C(T)) Method [28].

**Table 1:** Genes and primer sequences for RT-qPCR analysis.

No.	Genes	GenBank no.	Forward primer(5′ → 3′)	Reverse primer(5′ → 3′)
1	*CoFBA*1	JN017093.1	qQSM1F: ATCTTGGTGCCAGGGATTAG	qQSM1R: TCTCAGGTTCCACGATAGGC
2	*CoFBA*2	JX914588	qQSM2F: ATCATCTGCCAGGAGAATGG	qQSM2R: GTAAACCGCTGCAAGAACAC
3	*CoFBA*3	JX914589	qQSM3F: TTGCTGATTACACCCTCAAGCTC	qQSM3R: GATTGCCCACCAGACAAGAACAT
4	*CoFBA*4	JX914590	qQSM4F: GAGATTCTTCTTGATGGGGAT	qQSM4R: CATAGTGAGCGTGTATTTGGC
5	*CoGAPDH*	KC337052	qGAPDHF: CTACTGGAGTTTTCACCGA	qGAPDHR: TAAGACCCTCAACAATGCC
6	*CoACP*	EU717697	qACPF: ATTCAAGCAAAACCAGGCG	qACPR: CACACGAAATCCGAAAACG
7	*CoSAD*	KJ995982	qSADF: GTTCAAGTAACGCACTCCAT	qSADR: TTGCCAACATTTCTCCACAG
8	*CoFAD2*	KJ995981	qFAD2F: CCCAGCAACCAAACATGAAC	qFAD2R: GAATGAGCGGAGGAGAGAAC

### Oil Content and Fatty Acid Composition Analysis

Tea oil was extracted from seeds collected at eight developmental stages by Soxhlet extraction method according to the standard protocol of “Determination of crude fat in foods” (State Standard of the People's Republic of China, GB/T 14772-2008). Briefly, seed oil was extracted from the seeds with petroleum ether, dried and weighted. The extraction was repeated three times for each oil sample. Oil-yield rate  = (oil quantity/seed quantity)×100%. Seed lipids in the oil extract were converted to methyl esters by KOH-methanol solution and extracted with heptane. The organic phase containing lipids was transferred into a vial for GC analysis using a Gas Chromatograph (SHIMADZU GC-2014) following the basic methyl esterification method [18], [29]. GC analysis was performed using a FFAP capillary column (30 m×0.25 µm×0.25 mm) with the following conditions: 60°C →180°C (25°C/min,remain 1 min) →210°C (3°C/min, remain 1 min) →212°C (0.3°C/min,remain 1 min) →240°C (8°C/min,remain 2 min); inlet and detector temperature: 240°C; the split ratio: 1∶50; carrier gas flow velocity:N_2_ at 1.23 µL/min; compressed air: 400 µL/min; H_2_: 40 µL/min; sample injection: 1 µL. The oil content and fatty acid composition data were analyzed by SPSS Statistics 17.0 software (http://www.stathome.cn).

### Gray Correlation Analysis

The correlation coefficient between gene expression levels and oil content and fatty acid composition was obtained by the grey correlation analysis software (V2.1) [30], [31]. The oil content was used as reference series and the mRNA levels of the six genes were used as comparison series. The higher correlation coefficient between the mRNA levels and oil content/fatty acid composition means the more positive effect of the gene product on oil content/fatty acid composition. The basic idea of grey relational analysis is based on the similarity of sequence curve geometry. When the curves are closer, the correlation between the corresponding sequences is larger. When grey relational degree is bigger, the two factors have more consistent trends.

## Results

### Statistics of Transcriptome Sequencing Data

**Figure 1:**
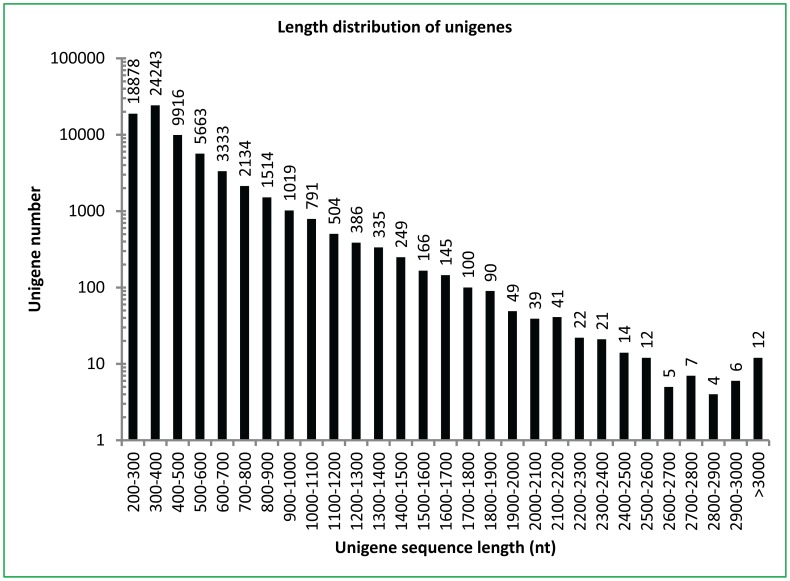
Length distribution of unigene sequences in tea oil seed transcriptome. The seed transcriptome was prepared using total RNA from the June and October seeds. The paired-end cDNA library was prepared according to Illumina's protocols and sequenced using the HiSeq 2000 platform. The distribution of unigenes was classified according to the length of unigenes.

**Figure 2:**
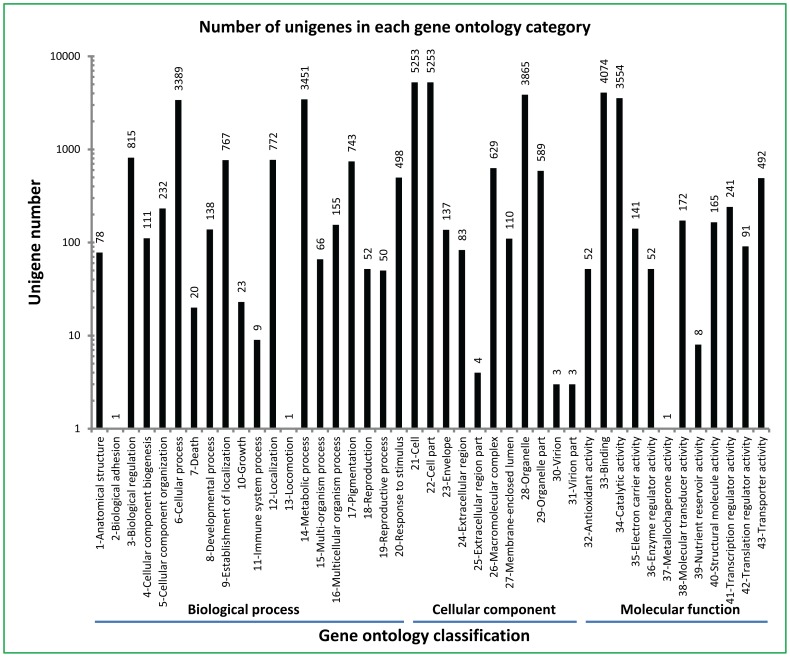
Gene ontology classification of unigenes (number of unigenes in each category) in tea oil seed transcriptome.

Transcriptome sequencing of the pair-ended cDNA libraries from two developmental stages of *Camellia oleifera* seeds collected on June 5 and October 24 generated 65,536 non-redundant unigenes with a total length of 24,154,817 nucleotides. The lengths of these unigenes were mainly distributed between 100 and500 nucleotides (Figure 1). These results indicated that our transcriptome sequencing was completed with high quality. All non-redundant unigenes could be classified into three GO (gene ontology) categories including molecular function, cellular component and biological process, which consisted of 43 functional groups (Figure 2).

### *CoFBA* Expression Analysis of the Transcriptome Data

Six FBA unigenes were identified from the transcriptome libraries constructed using two developmental stages of the oilseeds: Unigene 23265, Unigene 47982, Unigene 58261, Unigene 62390, Unigene 37785 and Unigene 63726. These *CoFBA* genes exhibited differential expression at different developmental stages of *Camellia oleifera* seeds (Table 2). Unigene 47982, Unigene 58261 and Unigene 62390 had False Discovery Rate (FDR)<0.01 and the differential expression was more than two folds between the peak and initial developmental stages (Table 2). These expression results suggest that the three FBA genes might play significant roles during tea oil biosynthesis process. In contrast, Unigene 37785 and Unigene 63726 genes did not express much at the initial developmental stage and their expression was down-regulated during the peak stage of oil accumulation (Table 2), suggesting that these two genes might have little effect on lipid biosynthesis in the seeds.

**Table 2:** *CoFBA* unigenes analysis.

Unigene No.	Expression (40 DAF)	Expression (180 DAF)	Log2	P value	False discovery rate	Expression pattern
23265	9	15	0.707819	0.250626	0.378055	Up-regulated
47982	47	215	2.163087571	0	0	Up-regulated
58261	301	1073	1.8035145603	0	0	Up-regulated
62390	17	45	1.374153	0.00046	0.002606	Up-regulated
37785	2	0	−5.90689	0.24221	0.409255	Down-regulated
63726	3	0	−6.49185	0.119834	0.258158	Down-regulated

### *CoFBA* Gene Expression Patterns during Seed Development

**Figure 3:**
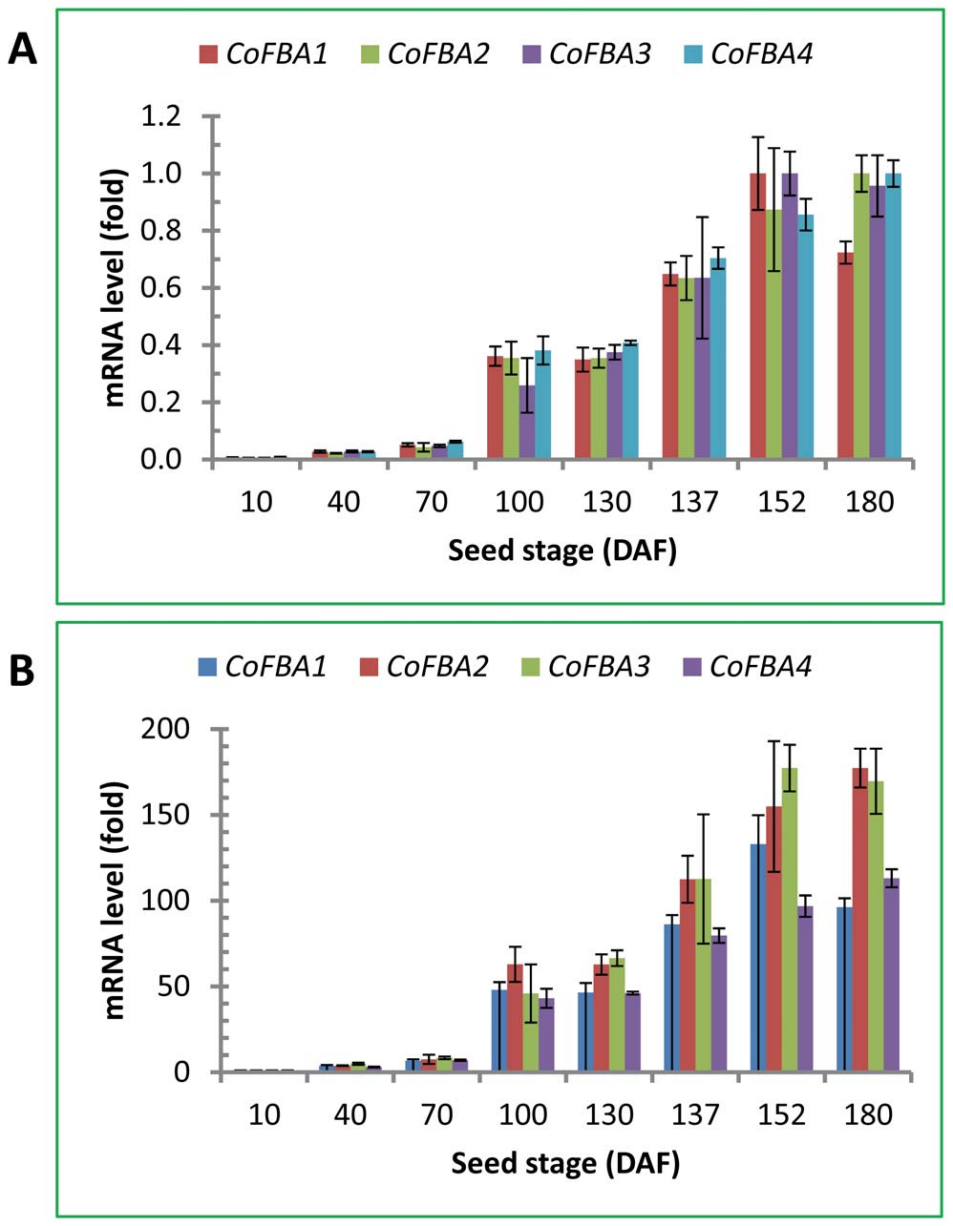
*CoFBA* mRNA levels in seeds at different developmental stages. *CoFBA* mRNA levels were quantified by RT-qPCR using total RNA from eight seed developmental stages (DAF, days after the end of flowering, corresponding to seeds collected on May 5, June 5, July 4, August 4, September 4, September 11, September 26 and October 24). RT-qPCR was performed in triplicates by SYBR Green qPCR assay using *CoGAPDH* gene as the reference gene. The mean and SD from triplicates are presented in the figure. (A) The highest value of relative mRNA levels was set at 1 fold. (B) The mRNA levels at 10 DAF was set at 1 fold.

The expression patterns of *CoFBA* genes were experimentally studied by RT-qPCR using RNA isolated from eight seed development stages (Figure 3). The four *FBA* genes including *CoFBA1* (Unigene 58261), *CoFBA2* (Unigene 47982), *CoFBA3* (Unigene 23265) and *CoFBA4* (Unigene 62390) showed similar expression patterns (Figure 3). Their mRNA levels were low but detectable at the initial oil synthesis stage (May 5 sample), gradually increased during the next two months, up-regulated sharply in early September and reached peak levels in late September (Figure 3). In more mature seeds collected in late October, the expression levels of *CoFBA1* and *CoFBA3* were slightly down-regulated, whereas those of *CoFBA2* and *CoFBA4* were still increased. These qPCR data (Figure 3) are in agreement with those of the transcriptome data described above (Table 2). Both lines of data support the roles of these genes in oil accumulation in tea oil seeds.

### *CoACP*, *CoSAD* and *CoFAD2* Gene Expression during Seed Development

**Figure 4:**
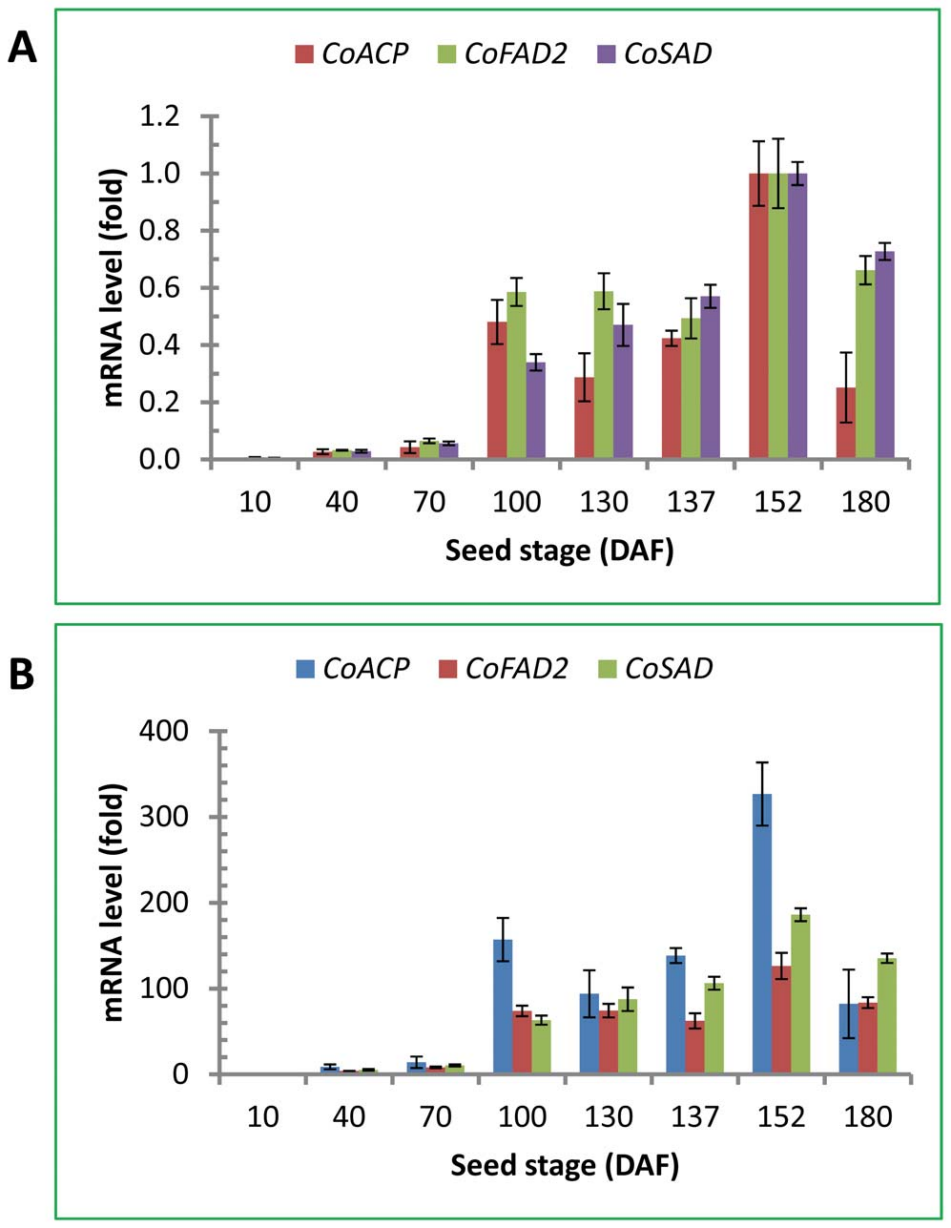
*CoACP*, *CoFAD2* and *CoSAD* mRNA levels in seeds at different developmental stages. The mRNA levels of these genes were quantified by RT-qPCR using total RNA from eight seed developmental stages (DAF, days after the end of flowering, corresponding to seeds collected on May 5, June 5, July 4, August 4, September 4, September 11, September 26 and October 24). RT-qPCR was performed in triplicates by SYBR Green qPCR assay using *CoGAPDH* gene as the reference gene. The mean and SD from triplicates are presented in the figure. (A) The highest value of relative mRNA levels was set at 1 fold. (B) The mRNA levels at 10 DAF was set at 1 fold.

RT-qPCR was also used to analyze the expression patterns of *CoACP*, *CoSAD* and *CoFAD2*, three genes directly involved in fatty acid metabolic pathway in the oilseeds. The common features of the expression of these three genes were that their relative expression levels in the seeds were extremely low during the early seed development but dramatically increased in early August and reached peak levels in late September followed by a sharp decline in late October (Figure 4). Some minor differences in mRNA levels among the three genes were noticed. During the period from May to October, *CoACP* mRNA levels exhibited two peaks of expression: a small peak in early August followed by small decline and a large peak in late September followed by sharp decline (Figure 4). *CoSAD* and *CoFAD2* exhibited only one peak of expression in late September during the same seed stages from May to October. *CoFAD2* mRNA levels were similar between early August and early September (Figure 4) but *CoSAD* mRNA levels were increased continually until peaked in late September (Figure 4).

### Oil Content and Fatty Acid Composition

**Figure 5:**
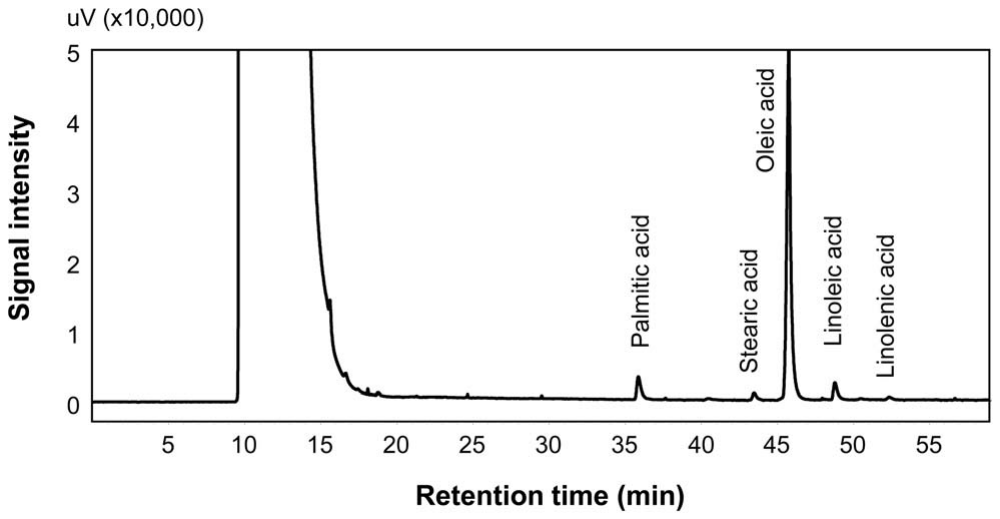
The chromatogram of fatty acids from tea oil. Tea oil was extracted from its seeds collected on October 24. Seed lipids were converted to methyl esters by KOH-methanol solution. Fatty acids were separated by GC.

**Figure 6:**
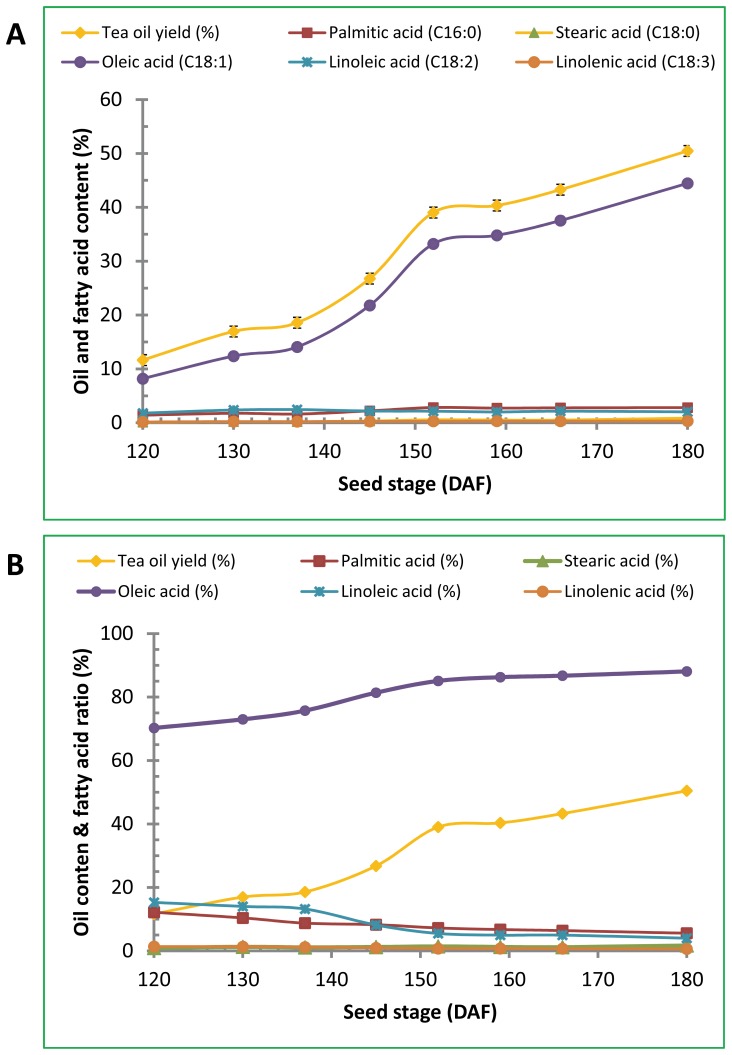
The oil content and fatty acid composition of *Camellia oleifera* seeds at different developmental stages. Tea oil seeds were collected at the days after the end of flowering (DAF) corresponding to August 25, September 4, September 11, September 19, September 26, October 3, October 10 and October 24. Tea oil was extracted from the seeds and converted to methyl esters by KOH-methanol solution. Fatty acids were separated and quantified by GC. (A) Tea oil and fatty acid content. Tea oil yield  =  (oil quantity/seed quantity)×100%. Fatty acid content  =  (tea oil yield/percentage of fatty acid)×100%. (B) Tea oil content and fatty acid composition. Fatty acid composition  =  (individual fatty acid peak under the curve/total fatty acid peaks under the curve)×100%.

Tea oil content and fatty acid composition were determined by Soxhlet extraction method and GC using the seeds collected from late August to late October. Seeds from these developmental stages were shown by qPCR to have active expression of *CoFBA CoACP*, *CoSAD* and *CoFAD2* genes (Figures 3 and 4). Representative chromatogram of fatty acid profiles from seeds collected in late October was shown in Figure 5. Oleic acid was the most abundant fatty acid in the seeds and accounted for 88% of the total fatty acids in the late stage of tea oil seeds, whereas other fatty acids accounted for only minor percentage of the tea oil. The developmental profile of oil accumulation in tea oil seeds showed that oil content was gradually increased from late August to late October and the degree of oil increases was smaller after late September, indicating that seeds began to mature after late September (Figure 6). The composition of fatty acids from the seeds collected at the eight developmental stages showed a remarkable correlation between the total oil content and oleic acid content in the seeds (Figure 6A). Oleic acid accounted for approximately 80% of the fatty acids in all stages of the seeds analyzed, and the other four fatty acids accounted for only 20% of the fatty acids in the seeds (Figure 6B). The general trends of the seed fatty acid composition were that the relative percentage of oleic acid increased a few percentages and other four fatty acids decreased accordingly in the seeds (Figure 6B). According to oil content, lipid synthesis was in the initial stage before early August. Oil composition could not be detected accurately in the seeds collected during these early stages by Soxhlet extraction method (data not shown).

### Correlation between *CoFBA, CoACP, CoFAD2* and *CoSAD* Gene Expression and Oil Content and Fatty Acid Composition

*CoACP, CoFAD2* and *CoSAD* are key enzymes for lipid synthesis. It is expected that the expression of these genes are closely related to oil content and/or fatty acid composition. The grey correlation analysis software evaluated the relevance between *CoFBA* mRNA levels, along with these three reference genes and oil content (Table 3). Relevance ranking showed that the order of correlation coefficient of the seven genes with tea oil content in the seeds were *CoSAD>CoFBA3>CoFBA1>CoACP>CoFBA4>CoFAD2>CoFBA2*. The correlation coefficient of *CoFBA* gene expression to fatty acid composition was less than 0.6. In contrast, there was a high correlation coefficient between *CoFAD2* expression levels and fatty acid composition in the seeds (Table 4).

**Table 3:** The relationship between gene expression levels and oil content at different developmental stages in tea oil seeds by Grey correlation degree analysis.

Factor	*CoSAD*	*CoFBA3*	*CoFBA1*	*CoACP*	*CoFBA4*	*CoFAD2*	*CoFBA2*
Correlation coefficient	0.97801	0.796754	0.75747	0.737026	0.729393	0.692832	0.690854
Order of correlation	1	2	3	4	5	6	7

**Table 4:** The relationship between gene expression levels and fatty acid composition at different developmental stages in tea oil seeds by Grey correlation degree analysis.

Factor	*CoFBA1*	*CoFBA2*	*CoFBA3*	*CoFBA4*	*CoSAD*	*CoACP*	*CoFAD2*
Palmitic acid	0.57335	0.557386	0.582001	0.56683	0.623204	0.568632	0.703032
Stearic acid	0.577173	0.559699	0.586809	0.569987	0.634386	0.571968	0.735296
Oleic acid	0.582968	0.56311	0.594212	0.574723	0.652996	0.576982	0.798968
Linoleic acid	0.564435	0.551781	0.571017	0.559349	0.599971	0.560765	0.646808
Linolenic acid	0.566443	0.55307	0.57465	0.561049	0.604891	0.562548	0.657668

## Discussion

Triacylglycerols are the major form of energy storage in eukaryotes. In addition, they serve as a reservoir of fatty acids for cellular membrane biogenesis and lead to obesity when excessively accumulated in adipose tissues [32]. Understanding plant TAG biosynthesis will facilitate creating oilseed crops with value-added properties [33]. However, choice of target genes for genetic engineering of plant oils is difficult because the plant oil is synthesized by at least 10 enzymatic steps and each step is catalyzed by multiple isozymes [17], [33], [34]. For example, tung oil biosynthetic pathway contains at least three genes coding for diacylglycerol acyltransferases (DGAT) [18], [34], three genes coding for fatty acid desaturases [35], and five genes coding for oleosins [36], [37].

*Camellia oleifera* (tea oil tree) provides widely used high quality cooking oil from the seeds. Tea oil is also very beneficial to human health. We have studied a number of genes in the oil biosynthetic pathway of tea oil to gain information for improving oil content and/or fatty acid composition in the seeds. In this study, we focused on *FBA* gene family which encodes a key enzyme that catalyzes the conversion of FBP into DHAP and G3P, two key intermediate metabolites for oil biosynthesis. We identified six forms of FBA from seed transcriptome and found the expression levels of four of them, along with three key oil biosynthesis genes, were well-correlated with oil content in developing tea oil seeds.

FBA is a key regulatory enzyme in the glycolytic pathway [38], which is located to the upstream of fatty acid biosynthesis pathway in oilseeds. In this study, we showed by RT-qPCR that the expression of *CoFBA* genes in tea oil seeds exhibited a trend of up-regulation before fruit enlargement stage and down-regulation slowly after reaching peak stage of oil accumulation, a pattern highly similar to that of *CoSAD* gene in developing tea oil seeds. The members of *CoFBA* gene family showed different expression patterns in the developmental seeds. Using grey correlation analysis software, the correlativity between expression levels of *CoFBA* genes and seed oil content/fatty acid composition was evaluated. Our results showed that the expression levels of *CoFBA* genes especially *CoFBA3* and *CoFBA1* genes were highly correlated with the amount (only behind *CoSAD* gene) but less with fatty acid composition of tea oil in the seeds. These results suggest that FBA may control the flux of carbohydrates and therefore play an important role in oil yield but has less effect on fatty acid composition in the oilseeds.

ACP, SAD and FAD2 are known enzymes/proteins in plant oil biosynthesis pathway. During the developmental process of *Camellia oleifera* seeds, the expression levels of these genes are coordinated with the oil content and fatty acid composition in the seeds [39]. ACP is located at the center of the fatty acid synthetase multi-enzyme complex and functions as a carrier of acyl by transferring acyl moiety from one enzymatic reaction to another [40]. SAD dehydrogenates the saturated fatty acid stearic acid (C18∶0) to form the monosaturated fatty acid oleic acid (C18∶1). Therefore, SAD may determine the total content of unsaturated oleic acid and the ratio of saturated stearic acid and unsaturated oleic acid [41], [42]. Since 80% of the fatty acids in tea oil is oleic acid, SAD may play an essential role in determining the amount of oleic acid and therefore the yield of tea oil in the seeds. This conclusion is supported by correlation analysis showing that the expression levels of *CoSAD* gene were highly correlated with the amount of tea oil in seeds (correlation coefficient was 0.97801). However, it is not clear from this analysis why the correlation between SAD mRNA levels and oleic acid itself was less significantly (0.652996, ranked second only to FAD2 with 0.798968). Under *FAD* gene family regulation, oleic acid is further dehydrogenated to form linoleic acid (C18∶2) and linolenic acid (C18∶3). In particular, FAD2 regulates the double-bond formation between 12^th^ and 13^th^ carbon atoms of oleic acid to produce linoleic acid. Therefore, FAD2 is considered an essential enzyme for determining the ratio of oleic acid and linoleic acid [43], [44]. This is supported by our data showing that *CoFAD2* mRNA levels were well-correlated with fatty acid composition in the oilseeds (ranked first among every fatty acid composition tested). However, only 2% of total fatty acids in tea oil are linoleci acid. Our correlation analysis is in agreement with the expectation that there could be a poor correlation between *CoFAD2* expression and oil content (ranked second to the last). Therefore, *CoFAD2* probably plays a minor role in the determination of oil content in tea oilseeds. Since plant oil is synthesized by at least 10 enzymatic steps and each step is catalyzed by multiple isozymes [17], [33], [34], it is important to identify other important genes critical for increasing tea oil content and fatty acid composition in future studies.

## Conclusions

This study identified multiple forms of *CoFBA* genes from seed transcriptome analysis, quantitatively evaluated the expression of four *CoFBA* genes in developing seeds, along with *CoACP, CoSAD* and *CoFAD2* genes, and analyzed the developmental profiles of oil content and fatty acid composition of tea oil. Correlation study indicated that the mRNA levels of *CoFBA* and *CoSAD* genes were positively correlated with oil content. The results suggest that *CoFBA* and *CoSAD* are important factors for determining tea oil yield because *CoFBA* gene controls the flux of key intermediates for oil biosynthesis and *CoSAD* gene controls the synthesis of oleic acid, the predominate fatty acid in tea oil. *CoFAD2* probably plays an important role in determining the composition of fatty acids in tea oil. This study suggests that tea oil yield could be improved by over-expression of *CoFBA* and *CoSAD* genes in transgenic plants.

## References

[1] Feas X, Estevinho LM, Salinero C, Vela P, Sainz MJ, et al (2013) Triacylglyceride, antioxidant and antimicrobial features of virgin Camellia oleifera, C. reticulata and C. sasanqua Oils. Molecules 18: 4573–4587. 23599015 10.3390/molecules18044573 PMC6270245

[2] Ye Y, Xing HT, Guo Y (2013) Hypolipidemic effect of a novel biflavonoid from shells of Camellia oleifera (Abel.). Indian J Exp Biol 51: 458–463. 23926694

[3] Zhuang RL (2008) Comprehensive utilization of tea-oil fruits. In: Tea-oil tree (*Camellia oleifera* Abel) of China. Beijing: Chinese Forestry Publish House. pp. 339–346.

[4] Chen L, Chen J, Xu H (2013) Sasanquasaponin from Camellia oleifera Abel. induces cell cycle arrest and apoptosis in human breast cancer MCF-7 cells. Fitoterapia 84: 123–129. 23164604 10.1016/j.fitote.2012.11.009

[5] Lee CP, Yen GC (2006) Antioxidant activity and bioactive compounds of tea seed (Camellia oleifera Abel.) oil. J Agric Food Chem 54: 779–784. 16448182 10.1021/jf052325a

[6] Cheng YT, Wu SL, Ho CY, Huang SM, Cheng CL, et al (2014) Beneficial Effects of Camellia Oil (Camellia oleifera Abel.) on Ketoprofen-Induced Gastrointestinal Mucosal Damage through Upregulation of HO-1 and VEGF. J Agric Food Chem 10.1021/jf404614k 24377395

[7] Ye Y, Guo Y, Luo YT (2012) Anti-Inflammatory and Analgesic Activities of a Novel Biflavonoid from Shells of Camellia oleifera. Int J Mol Sci 13: 12401–12411. 23202905 10.3390/ijms131012401 PMC3497279

[8] Ye Y, Guo Y, Luo YT, Wang YF (2012) Isolation and free radical scavenging activities of a novel biflavonoid from the shells of Camellia oleifera Abel. Fitoterapia 83: 1585–1589. 22982330 10.1016/j.fitote.2012.09.006

[9] Guo J, Tan X, Wang W, Zhang D, Zhang L, et al (2010) Isolation and cloning of full-length cDNA of FatB genes from Camellia oleifera and its sequence analysis. Journal of Central South University of Forestry & Technology 30: 66–75.

[10] Tan X, Hu F, Xie L, Shi M, Zhang D, et al (2006) Construction of EST library and analysis of main expressed genes of *Camellia oleifera* seeds. Scientia Silvae Sinicae 42: 43–48.

[11] Tan X, Wang W, Liu Z, Zhang D, Chen H, et al (2008) Cloning of full-length cDNAs from acyl carrier protein gene of *Camellia oleifera* and its sequence analysis. Journal of Central South University of Forestry & Technology 28: 8–14.

[12] Tan X, Jang Y, Wang B, Zhang L (2010) Cloning and sequence analysis of BC gene from *Camellia oleifera* . Journal of Central South University of Forestry & Technology 30: 1–9.

[13] Zhang D, Tan X, Chen H, Zeng Y, Jang Y, et al (2008) Full-length cDNA Cloning and Bioinformatic Analysis of Camellia oleifera SAD. Scientia Silvae Sinicae 44: 155–159.

[14] Zhu F, Chen H, Tan X, Liu K, Wang J (2012) Cloning and sequence analysis of full-length cDNA of LOX gene from *Camellia oleifera* . Journal of Central South University of Forestry & Technology 32: 51–57.

[15] Rutter WJ (1964) EVOLUTION OF ALDOLASE. Fed Proc 23: 1248–1257. 14236133

[16] Vigeolas H, Waldeck P, Zank T, Geigenberger P (2007) Increasing seed oil content in oil-seed rape (Brassica napus L.) by over-expression of a yeast glycerol-3-phosphate dehydrogenase under the control of a seed-specific promoter. Plant Biotechnol J 5: 431–441. 17430545 10.1111/j.1467-7652.2007.00252.x

[17] Cao H (2011) Structure-function analysis of diacylglycerol acyltransferase sequences from 70 organisms. BMC Res Notes 4: 249. 21777418 10.1186/1756-0500-4-249 PMC3157451

[18] Cao H, Shockey JM, Klasson KT, Chapital DC, Mason CB, et al (2013) Developmental regulation of diacylglycerol acyltransferase family gene expression in tung tree tissues. PLoS ONE 8: e76946. 24146944 10.1371/journal.pone.0076946 PMC3795650

[19] Thelen JJ, Ohlrogge JB (2002) Metabolic engineering of fatty acid biosynthesis in plants. Metab Eng 4: 12–21. 11800570 10.1006/mben.2001.0204

[20] Tan X, Chen H, Zhang D, Zeng Y, Li W, et al (2008) Cloning of Full-length cDNA of FAD2 Gene from *Camellia oleifera* . Scientia Silvae Sinicae 44: 70–75.

[21] Grabherr MG, Haas BJ, Yassour M, Levin JZ, Thompson DA, et al (2011) Full-length transcriptome assembly from RNA-Seq data without a reference genome. Nat Biotechnol 29: 644–652. 21572440 10.1038/nbt.1883 PMC3571712

[22] Conesa A, Gotz S, Garcia-Gomez JM, Terol J, Talon M, et al (2005) Blast2GO: a universal tool for annotation, visualization and analysis in functional genomics research. Bioinformatics 21: 3674–3676. 16081474 10.1093/bioinformatics/bti610

[23] Iseli C, Jongeneel CV, Bucher P (1999) ESTScan: a program for detecting, evaluating, and reconstructing potential coding regions in EST sequences. Proc Int Conf Intell Syst Mol Biol 138–148. 10786296

[24] Audic S, Claverie JM (1997) The significance of digital gene expression profiles. Genome Res 7: 986–995. 9331369 10.1101/gr.7.10.986

[25] Mortazavi A, Williams BA, McCue K, Schaeffer L, Wold B (2008) Mapping and quantifying mammalian transcriptomes by RNA-Seq. Nat Methods 5: 621–628. 18516045 10.1038/nmeth.1226 PMC13303166

[26] Wang B, Tan X, Chen Y, Zeng Y (2012) Molecular cloning and expression analysis of two calmodulin genes encoding an idential protein from *Camellia oleifera* . Pak J Bot 44: 961–968.

[27] Cao H, Shockey JM (2012) Comparison of TaqMan and SYBR Green qPCR methods for quantitative gene expression in tung tree tissues. J Agric Food Chem 60: 12296–12303. 23176309 10.1021/jf304690e

[28] Livak KJ, Schmittgen TD (2001) Analysis of relative gene expression data using real-time quantitative PCR and the 2(-Delta Delta C(T)) Method. Methods 25: 402–408. 11846609 10.1006/meth.2001.1262

[29] Wang X, Cao Y, Zhang L, Chen Y (2012) Analysis of the Fatty Acids Composition of *Camellia* in Different Growth Stages. Chinese Agricultural Science Bulletin 28: 76–80.

[30] Liu S, Cai H, Yang Y, Cao Y (2013) Advance in grey incidence analysis modeling. System Engineering-Theory & Practice 33: 2041–2046.

[31] Zhang L, Jia B, Tan X, Thammina CS, Long H, et al (2014) Fatty acid profile and unigene-derived simple sequence repeat markers in tung tree (Vernicia fordii). PLoS ONE 9 In press.. 10.1371/journal.pone.0105298 PMC4148264 25167054

[32] Farese RV Jr, Walther TC (2009) Lipid droplets finally get a little R-E-S-P-E-C-T. Cell 139: 855–860. 19945371 10.1016/j.cell.2009.11.005 PMC3097139

[33] Dyer JM, Stymne S, Green AG, Carlsson AS (2008) High-value oils from plants. Plant J 54: 640–655. 18476869 10.1111/j.1365-313X.2008.03430.x

[34] Shockey JM, Gidda SK, Chapital DC, Kuan JC, Dhanoa PK, et al (2006) Tung tree DGAT1 and DGAT2 have nonredundant functions in triacylglycerol biosynthesis and are localized to different subdomains of the endoplasmic reticulum. Plant Cell 18: 2294–2313. 16920778 10.1105/tpc.106.043695 PMC1560902

[35] Dyer JM, Chapital DC, Kuan JC, Mullen RT, Turner C, et al (2002) Molecular analysis of a bifunctional fatty acid conjugase/desaturase from tung. Implications for the evolution of plant fatty acid diversity. Plant Physiol 130: 2027–2038. 12481086 10.1104/pp.102.010835 PMC166714

[36] Long H, Tan X, Chen H, Zhang L, Hu J (2010) Cloning and sequence analysis of full-length cDNAs encoding oleosins from Vernicia fordii. Journal of Central South University of Forestry and Technology 30: 31–38.

[37] Cao H, Zhang L, Tan X, Long H, Shockey JM (2014) Identification, Classification and Differential Expression of Oleosin Genes in Tung Tree (*Vernicia fordii*). PLoS ONE 9. 10.1371/journal.pone.0088409 PMC3916434 24516650

[38] Haake V, Geiger M, Walch-Liu P, Of-áEngels C, Zrenner R, et al (1999) Changes in aldolase activity in wild-type potato plants are important for acclimation to growth irradiance and carbon dioxide concentration, because plastid aldolase exerts control over the ambient rate of photosynthesis across a range of growth conditions. The Plant Journal 17: 479–489.

[39] Zeng Y, Tan X, Zhang D, Chen H, Zeng X, et al (2014) Research on regulation about control oil synthesis by key genes in fatty acid metabolic pathway of *Camellia oleifera* . Journal of the Chinese Cereals and Oils Association 29: 26–29.

[40] Prescott DJ, Elovson J, Vagelos PR (1975) Acyl carrier protein synthetase. Methods Enzymol 35: 95–101. 1091814 10.1016/0076-6879(75)35143-4

[41] Kachroo A, Shanklin J, Whittle E, Lapchyk L, Hildebrand D, et al (2007) The Arabidopsis stearoyl-acyl carrier protein-desaturase family and the contribution of leaf isoforms to oleic acid synthesis. Plant Mol Biol 63: 257–271. 17072561 10.1007/s11103-006-9086-y

[42] Yukawa Y, Takaiwa F, Shoji K, Masuda K, Yamada K (1996) Structure and expression of two seed-specific cDNA clones encoding stearoyl-acyl carrier protein desaturase from sesame, Sesamum indicum L. Plant Cell Physiol 37: 201–205. 8665096 10.1093/oxfordjournals.pcp.a028932

[43] Los DA, Murata N (1998) Structure and expression of fatty acid desaturases. Biochim Biophys Acta 1394: 3–15. 9767077 10.1016/s0005-2760(98)00091-5

[44] Shanklin J, Cahoon EB (1998) DESATURATION AND RELATED MODIFICATIONS OF FATTY ACIDS1. Annu Rev Plant Physiol Plant Mol Biol 49: 611–641. 15012248 10.1146/annurev.arplant.49.1.611

